# Slow oscillating transcranial direct current stimulation during non-rapid eye movement sleep improves behavioral inhibition in attention-deficit/hyperactivity disorder

**DOI:** 10.3389/fncel.2015.00307

**Published:** 2015-08-11

**Authors:** Manuel T. Munz, Alexander Prehn-Kristensen, Frederieke Thielking, Matthias Mölle, Robert Göder, Lioba Baving

**Affiliations:** ^1^Department of Child and Adolescent Psychiatry and Psychotherapy, Center for Integrative Psychiatry, School of Medicine, Christian-Albrechts-University KielKiel, Germany; ^2^Institute of Neuroendocrinology, School of Medicine, University of LübeckLübeck, Germany; ^3^Department of Psychiatry and Psychotherapy, Center for Integrative Psychiatry, School of Medicine, Christian-Albrechts-University KielKiel, Germany

**Keywords:** slow oscillations, attention deficit/hyperactivity disorder, prefrontal cortex, behavioral inhibition, transcranial direct-current stimulation

## Abstract

**Background:** Behavioral inhibition, which is a later-developing executive function (EF) and anatomically located in prefrontal areas, is impaired in attention-deficit and hyperactivity disorder (ADHD). While optimal EFs have been shown to depend on efficient sleep in healthy subjects, the impact of sleep problems, frequently reported in ADHD, remains elusive. Findings of macroscopic sleep changes in ADHD are inconsistent, but there is emerging evidence for distinct microscopic changes with a focus on prefrontal cortical regions and non-rapid eye movement (non-REM) slow-wave sleep. Recently, slow oscillations (SO) during non-REM sleep were found to be less functional and, as such, may be involved in sleep-dependent memory impairments in ADHD.

**Objective:**By augmenting slow-wave power through bilateral, slow oscillating transcranial direct current stimulation (so-tDCS, frequency = 0.75 Hz) during non-REM sleep, we aimed to improve daytime behavioral inhibition in children with ADHD.

**Methods:** Fourteen boys (10–14 years) diagnosed with ADHD were included. In a randomized, double-blind, cross-over design, patients received so-tDCS either in the first or in the second experimental sleep night. Inhibition control was assessed with a visuomotor go/no-go task. Intrinsic alertness was assessed with a simple stimulus response task. To control for visuomotor performance, motor memory was assessed with a finger sequence tapping task.

**Results:** SO-power was enhanced during early non-REM sleep, accompanied by slowed reaction times and decreased standard deviations of reaction times, in the go/no-go task after so-tDCS. In contrast, intrinsic alertness, and motor memory performance were not improved by so-tDCS.

**Conclusion:** Since behavioral inhibition but not intrinsic alertness or motor memory was improved by so-tDCS, our results suggest that lateral prefrontal slow oscillations during sleep might play a specific role for executive functioning in ADHD.

## Introduction

Attention-deficit and hyperactivity disorder (ADHD) represents a neurodevelopmental disorder with a prevalence of 3–6% and is characterized by “age-inappropriate levels of hyperactivity, impulsivity, and inattention” (American Psychiatric Association, 2000; Polanczyk et al., 2007). Deficits in behavioral inhibition (Barkley, 1997) have been described as a primary deficit in ADHD (Willcutt et al., 2005; Adams et al., 2008). Behavioral inhibition refers to three interrelated processes: the inhibition of the initial prepotent response to a stimulus, cessation of an ongoing response, and interference control. In this model, inhibition is linked to higher order “executive functions.” Similarly, deficits in EF have been suggested to be crucial hallmarks of ADHD (Pennington and Ozonoff, 1996; Willcutt et al., 2005). Neural networks underlying EF are anatomically located in different subregions of the prefrontal cortex (PFC) and exert their function via multiple connections with the sensory and motor cortices, the basal ganglia, and the cerebellum (Middleton and Strick, 2000; Barbas and Zikopoulos, 2007). The behavioral observation that children with ADHD behave like younger children is believed to result from a delay in structural and functional brain maturation, particularly in the PFC (Shaw et al., 2006, 2007, 2008; Rubia, 2007; Shaw and Rabin, 2009). Also, EEG slow-wave activity (SWA, ranging from 0.5 to 4.5 Hz in the literature) undergoes substantial changes during development and has been shown to accompany cortical maturation processes (Kurth et al., 2010; Buchmann et al., 2011).

Sleep problems are frequently reported in ADHD (Yoon et al., 2012), and treatment of sleep problems can improve cognitive functioning and reduce ADHD-like symptomatology (Cortese et al., 2009; Konofal et al., 2010). Despite the close relationship between sleep and cognitive symptoms, findings of macroscopic sleep deviations and their functional implications in ADHD are inconsistent (Cortese et al., 2006; Gau et al., 2007; Konofal et al., 2010; Yoon et al., 2012). Recent studies focusing on micro-architecture and sleep function propose that SWA in prefrontal regions may represent a promising polysomnographic marker in ADHD. Using high definition EEG, SWA has recently been found to be increased over central areas, whereas controls displayed a power maximum over frontal areas (Ringli et al., 2013a). The pattern found in ADHD is typical for earlier developmental stages, corresponding to the maturational delay hypothesis of ADHD (Rubia, 2007). On a functional level, slow oscillations (SO), ultra-slow, and highly synchronized oscillatory activity (<1 Hz) assumed to originate in the PFC (Massimini et al., 2004; Murphy et al., 2009), displayed impaired functionality and seems to be involved in deviant information processing with weaker declarative sleep-associated memory consolidation in ADHD (Prehn-Kristensen et al., 2011a,b, 2013). SO represent a hallmark characteristic of deep non-REM sleep, and their initial amplitude and slope of SO during nighttime sleep is thought to represent restorative processes (Tononi and Cirelli, 2006; Heller, 2013).

EF in children depend on both sleep quantity and efficiency (Steenari et al., 2003); a growing body of literature suggests that beneficial (non-REM) sleep parameters are related to daytime executive functioning in ADHD (Gruber and Sadeh, 2004; Durmer and Dinges, 2005; Sadeh et al., 2006; Gruber et al., 2007, 2011). Prefrontal functions seem to be particularly vulnerable to sleep impairment (Killgore, 2010). However, the mechanisms, which determine how altered sleep parameters relate to specific daytime functioning deficits in general and in ADHD, remain elusive (Yoon et al., 2012; Turnbull et al., 2013). By combining daytime deficits in behavioral inhibition or executive functioning, functional, and structural alterations in prefrontal regions, and altered prefrontal slow oscillatory activity and function during sleep in ADHD, we aimed to investigate the functional significance of non-REM slow oscillatory activity in relation to intrinsic alertness, behavioral inhibition, and executive functions, respectively.

Slow oscillating transcranial, direct-current stimulation (so-tDCS, frequency = 0.75 Hz) has previously been shown to interact with physiological slow oscillatory activity. At the behavioral level, stimulation supported declarative memory processes in healthy and patient populations, including compensation of sleep-dependent memory deficits in ADHD (Marshall et al., 2006; Göder et al., 2013; Prehn-Kristensen et al., 2014). Using so-tDCS to induce slow oscillatory activity over lateral prefrontal regions in children with ADHD, we hypothesized that slow oscillatory activity would be enhanced and that inhibition control would improve.

## Materials and methods

### Subjects

A total of 14 children meeting the criteria for ADHD according to DSM-IV-R (American Psychiatric Association, 2000) were included in the study (8 combined subtype, 6 inattentive subtype; mean age 12.3 ± 1.39 years, range 10–14 years). Patients previously diagnosed with ADHD were recruited via the out-patient unit of the Department of Child and Adolescent Psychiatry and Psychotherapy of the Center for Integrative Psychiatry in Kiel, Germany, or by newspaper advertisements. The diagnosis was determined using a German version of the Revised Schedule for Affective Disorders and Schizophrenia for School-Age Children: Present and Lifetime Version (K-SADS-PL) (Delmo et al., 2000). Additionally, the Child Behavior Checklist (CBCL) (Achenbach, 1991), a standard questionnaire, was filled out by parents in order to screen for additional psychiatric symptoms. Intelligence was estimated using the first part of the Culture Fair Intelligence Test 20-Revised version CFT-20R (Weiß, 2006). The mean intelligence score was 102 ± 9.44, ranging between 81 and 117. Sleep disorders were assessed using the Sleep-Self-Report questionnaire (Schwerdtle et al., 2010). Patients were excluded, if they scored above 25 points (cut-off).

Patients were also excluded, if there was a history of any neurological, endocrinological, immunological disorder (according to self-reports), or any psychiatric condition other than ADHD or comorbid oppositional defiant disorder (3 patients) or conduct disorder (2 patients). Four patients were free of medication, and 10 patients had previously been using methylphenidate but discontinued medication 48 h prior to each of the experimental conditions. Patients were excluded, if they were on any other medication.

The study was approved by the ethics committee of the Medical Faculty of the University in Kiel and followed the declaration of Helsinki. All children and their parents give written informed consent. Patients received a voucher for their participation.

### Go/no-go task (behavioral inhibition)

We used the subtest *Ablenkbarkeit* (English: “distractibility”) from the computerized version of the “Kinder–Testbatterie für Aufmerksamkeitsprüfung” (KiTAP) (Zimmermann et al., 2005). In a randomized order, either a go-stimulus (sad ghost, 40 trials) or a no-go-stimulus (smiling ghost, 40 trials) appeared on a computer screen. The ghost was displayed for 200 ms, and the interstimulus interval was variable. Participants were instructed to press a button only when the sad ghost appeared but to refrain from responding when the smiling ghost was displayed. Additionally, in 50% of the trials an additional visual distractor was presented (e.g., a cat) 400 ms before the stimulus appeared, which the participants were instructed to ignore. The total duration of the task was 190 s. Dependent variables were the number of correct trials, the number of commission errors, the number of omissions, reaction times of correct trials, and the variability of reaction times of correct trials. Prior to the main task, a training task was conducted to ensure that the participants understood the instructions.

### Psychometric and cognitive control tasks

In order to control for possible effects of daytime and stimulation on intrinsic alertness, we used the subtest *Alertness* which is also a subtest of the computerized version of the “Kinder–Testbatterie für Aufmerksamkeitsprüfung,” KiTAP (Zimmermann et al., 2005). Here, a witch repeatedly appeared in a centrally located window on the screen. Participants were instructed to press a single response button as soon as they saw the witch. In total, there were 30 trials. The task duration was 110 s, and the interstimulus interval was variable. Prior to the task, a training task was conducted in order to ensure that the participants had understood the instructions. Dependent variables were reaction times (RT) and the variability of reaction times (vRT).

As a visuomotor control variable, motor memory performance was assessed with a modified version of the finger sequence tapping task (Walker et al., 2003a; Wilhelm et al., 2008). This task requires subjects to press four numeric keys on a keyboard using the fingers of their non-dominant hand. Four boxes were displayed horizontally on the computer screen, and a white star repeatedly appeared in one of the four boxes. The subjects were instructed to press the corresponding key as quickly and as accurately as possible. Once a key was pressed, another star appeared in one of the boxes, and the corresponding key had to be pressed. One trial lasted 30 s, followed by a 30-s pause. At the end of each trial, a visual feedback (total number of key presses and number of correct key presses) was given. There was a fixed sequence of five elements (e.g., 2–4–1–3–2) underlying the order of the required key presses; the subjects were not, however, informed about this, in order to keep explicitness of the memory trace low. Two parallel sequences were used and randomized over the experimental conditions. During the learning sessions, participants completed 12 contiguous trials. Participants were told that they would have to complete the same task the next morning. At retest, three trials were conducted with the exact same sequence. The number of correct key presses, the number of total key presses, and the RT were recorded. Accuracy was calculated as the ratio of correct key presses relative to the total number of key presses (Walker et al., 2003b).

### so-tDCS stimulation and EEG data collection

Slow oscillations were introduced with two battery-driven, constant-current stimulators (neuroconn, Ilmenau, Germany). Two Ag/AgCl sintered skin electrodes (13 mm outer diameter; 8 mm inner diameter: 0.503 cm^2^ area) were placed bilaterally at frontolateral locations (F3, F4 according to the international 10–20 system, anodal polarization) and at the ipsilateral mastoids. Frontal electrodes were affixed by adhesive EC2 paste (Grass, USA); the mastoid electrodes were filled with chloride, abrasive electrolyte paste, and affixed by adhesive washers (Easycap, Germany). Field potentials were induced by applying sinusoidal currents (maximum current density: 0.517 mA/cm^2^) oscillating at a frequency of 0.75 Hz. The current strength at the anodal electrodes ranged from 0 to 250 μA. The maximum current density was 0.497 mA/cm^2^ (250 μA/0.503 cm^2^). Four min after subjects had entered the non-REM sleep stage 2 for the first time, stimulation was started and applied during five intervals with a duration of 5 min each and separated by 1-min intervals without stimulation. In the sham condition, the electrodes were applied just like in the stimulation condition but the stimulator remained off. The stimulation protocol was adopted from Marshall et al. (2006). As a small modification, the shape of our applied oscillating potentials was changed from on/off with rising and falling slopes to sinusoidal in order to achieve a close imitation of endogenous slow oscillations and to minimize sensations to the participants.

All EEG data were collected in the sleep laboratory of the Center for Integrative Psychiatry in Kiel using a 16-channel somnography device (Somnomedics, Randesacker, Germany). Electrodes were placed according to the international 10–20 system at the locations Fz, C3, C4, P3, P4, Pz, Oz, and mastoids and referenced to Cz with a ground placed at AFz. The EOG was recorded from the lower right and upper left canthi. The EEG and EOG were sampled at 128 Hz (band-pass filter: 0.2–75 Hz). The EMG was recorded from the chin and from the left and right lower legs at 256 Hz (0.2–128 Hz). Additionally, nasal air flow, nasal air pressure, and chest motion were monitored with a thermistor and a piezoelectric band, respectively. The raw data were visually scored (30 s epochs, American Academy of Sleep Medicine, 2007), and NREM sleep stages were defined according to Rechtschaffen and Kales (1968). Time in bed was calculated as the interval between lights out and lights on. Total sleep time was calculated as the minutes spent asleep during time in bed. Sleep efficiency was calculated as the percentage of time asleep compared to the time spent in bed.

### Experimental design and procedures

All participants underwent two experimental conditions consisting of a night in the sleep laboratory with neurocognitive testing the evening before and the morning after. Between the experimental nights, there was an interval of 1 week. Stimulation and sham-stimulation, respectively, and parallel versions of a motor memory task were pseudo-randomized over the experimental sessions. Experimenters and subjects were blind to stimulation. All subjects slept in the same room during both experimental nights. The participants arrived at the sleep laboratory at 7 p.m. After dinner, electrodes were mounted. At 8 p.m the cognitive tasks, including control tasks, were conducted in the following order: *Alertness* (KiTAP), encoding of a declarative memory task, encoding of the motor memory task. Thereafter, participants went to bed and were asked to begin sleeping while the experimenter left the laboratory. After participants fell asleep, so-tDCS or sham stimulation was applied. The next morning, patients were awakened at 6:30 a.m., and electrodes were removed. After breakfast, the cognitive testing began at 7:30 a.m. After awakening, an interval of 60 min was maintained so that the results would not be influenced by post-sleep inertia. In the morning session, the tests were conducted in the following order: *Alertness* (KiTAP), retrieval of declarative memory task (results are reported elsewhere, Prehn-Kristensen et al., 2014), retrieval of motor memory task, and go/no-go task (*Ablenkbarkeit* KiTAP).

Prior to each experimental night, patients were familiarized with the sleep laboratory during an adaptation night, during which we also screened for sleep disturbances and sleep-related breathing disorders. In the experimental nights, EEG, EOG, and chin EMG data were collected.

### Statistical analyses

Statistical analysis was performed with SPSS, version 21, for Windows.

Regarding inhibition control correct responses, errors, omissions, RT as well as reaction time standard deviations were analyzed each by a repeated measured analysis of variance (ANOVA) with the within factors CONDITION (stimulation vs. sham) and DISTRACTION (without vs. with).

With respect to basic attention performance, correct responses, omissions, reaction times, and reaction time, standard deviations were all analyzed by a repeated measures ANOVA with the within factors CONDITION (stimulation vs. sham) and SESSION (evening vs. morning).

For the motor memory task, RT, and accuracy were analyzed by a repeated measures ANOVA with the within factors CONDITION (stimulation vs. sham) and SESSION (learning vs. recall). Differences in single means were tested by paired *t*-tests.

Quantitative sleep parameters from the stimulation and sham night were compared using paired *t*-tests. Slow oscillation power in the 1-min post-stimulation intervals and the equivalents in the sham condition were calculated using a 2 × 8 ANOVA with the within factors Condition (stimulation vs. sham) and Electrode Position (Fz, Cz, C3, C4, Pz, P3, P4, Oz). Slow oscillation power in S4 during the 1 min-intervals was analyzed with an ANOVA with the within factors CONDITION (stimulation vs. sham) and POSITION (Fz, Cz, C3, C4, Pz, P3, P4 and Oz).

## Results

None of the patients reported any side effects during or after transcranial stimulation.

### Sleep parameters

We found no differences in quantitative measurements of sleep stages, total time in bed, total sleep time, or sleep efficiency between the stimulation and sham conditions (*p* > 0.4). Since stimulation produced saturation artifacts, the 25 min of stimulation and equivalent epochs during sham stimulation were excluded from the analysis of sleep stages. For the calculation of time in bed (TIB), total sleep time (TST), and sleep efficiency, stimulation epochs were counted as sleep, since all participants were asleep before and after each stimulation epoch. Sleep parameters are provided in Table 1.

**Table 1:** **Sleep parameters**.

	**STIM**	**SHAM**	***p*-values**
	**Mean ± *SD***	**Mean ± *SD***	
TIB (min)	555.9 ± 16.4	551.2 ± 14.4	0.48
TST (Min)	476.6 ± 64.4	472.3 ± 33.4	0.785
Sleep efficiency (%)	85.4 ± 10.4	85.4 ± 5.4	0.998
**SLEEP STAGES (%)**			
REM	20.9 ± 3.2	21.7 ± 3.4	0.469
S1	6.3 ± 2.4	6.3 ± 2.4	0.934
S2	46.6 ± 7.5	46.0 ± 6	0.622
S3	11.1 ± 2.7	11.5 ± 3	0.486
S4	15.2 ± 6.8	14.5 ± 5	0.62
S3/4	26.2 ± 8.2	26.0 ± 8.2	0.895

*STIM, stimulation; SHAM, sham stimulation; SD, standard deviation; TIB, time in bed; TST, total sleep time; REM, rapid eye movement*.

### Slow oscillation power

There was a main effect of CONDITION [*F*_(1, 13)_ = 9.615; *p* = 0.008] and a main effect of POSITION [*F*_(7, 91)_ = 15.453; *p* < 0.001]. However, there was no CONDITION × POSITION interaction. As such, the SO power averaged over all electrodes was higher in the 1 min post-stimulation intervals than in equivalent intervals in the sham condition during non-REM sleep stage 4, indicating an increase in SO power due to so-tDCS.

### Go/no-go task

**Figure 1:**
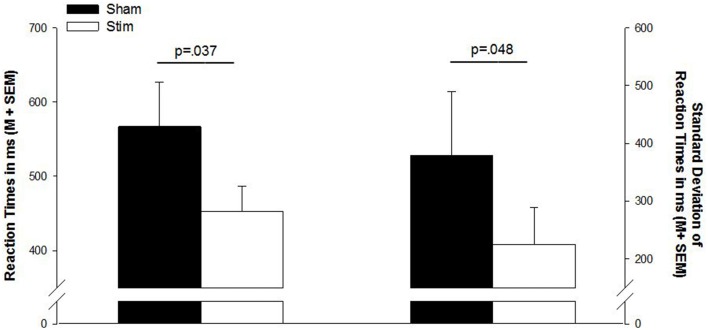
**Mean reaction times and standard deviation of reaction times in the go/no-go task**. Sham, sham stimulation (black bars); Stim, stimulation (white bars); M, mean; SEM, standard error of the mean.

There were no differences in the number of correct responses, number of false responses, or omission errors (*p* > 0.1). However, as indicated by a main effect for CONDITION [*F*_(1, 13)_ = 2.32; *p* = 0.037], the RT were shorter after a night of stimulation than after sham treatment. Also, the standard deviations of the reaction times were smaller in the stimulation condition [*F*_(1, 13)_ = 2.18; *p* = 0.048]. Reaction times and standard deviations of reaction times are depicted in Figure 1. Table 2 gives a detailed overview over the results of the go/no-go task.

**Table 2:** **Results of the go/no-go task**.

	**STIM**	**SHAM**	***t*_(13)_**	***p*-values**
	**Mean ± *SD***	**Mean ± *SD***		
RT (ms)	453.2 ± 131.3	566.9 ± 234.1	2, 32	**0.037**
SD of RT (ms)	225.2 ± 246.9	379.4 ± 425.3	2, 18	**0.048**

*STIM, stimulation; SHAM, sham stimulation, SD, standard deviation; RT, reaction time; bold numbers indicate significant mean differences*.

### Intrinsic alertness

There was no main effect of CONDITION (*p* > 0.3) and no main effect of SESSION (*p* > 0.5). Also, there was no CONDITION × SESSION interaction (*p* > 0.5). The ANOVA of standard deviations of reaction times did not reveal any main effect of CONDITION (*p* > 0.6) nor a main effect of SESSION (*p* = 0.095). Also, there was no CONDTITION × SESSION interaction (*p* > 0.9). Exploratory, paired-sample *t*-tests did not indicate any significant mean differences (*p* > 0.3). Means and standard deviations are shown in Table 3.

**Table 3:** **Results of the control tasks “Alertness” and “Motor memory”**.

			**Stim**	**Sham**
			**Mean ± *SD***	**Mean ± *SD***
Alertness	RT (ms)	Learning Recall	314.21 ± 56.0 309.64 ± 51.8	303.93 ± 36.0 302.40 ± 44.3
Motor memory	RT (ms)	Learning Recall	427.70 ± 125.8 356.35 ± 97.3	412.89 ± 108.9 349.13 ± 100.7
	Accuracy (%)	Learning Recall	94.2 ± 1.9 94.8 ± 1.8	94.1 ± 1.3^*^ 96.4 ± 0.9^*^

*Stim, Stimulation; Sham, sham stimulation; SD, standard deviation; ms, milliseconds*.

* *significant increase in accuracy by means of an exploratory t-test (p = 0.004)*.

### Motor memory task

Mean reaction times of the last three trials during the learning session and the mean reaction times of the first three trials in the recall session were analyzed. There was a main effect of SESSION [*F*_(1, 13)_; *p* < 0.001] but no main effect of CONDITION or CONDITION × SESSION interaction (*p* > 0.4), indicating a significant overnight gain in speed irrespective of stimulation. Mean reaction times and standard deviations are provided in Table 3.

Accuracy was calculated for the last three trials of the learning session and the first three trials of the recall session. There was no main effect of CONDITION or SESSION (*p* > 0.2) and no CONDITION × SESSION interaction (*p* > 0.4). However, an exploratory paired-sample *t*-test revealed a significant sleep-associated gain in accuracy without stimulation (*p* = 0.004) which was not present after stimulation (*p* = 0.819). Accuracy scores are depicted in Table 3.

## Discussion

In the present study, we used transcranial direct current stimulation to introduce slow oscillations during deep sleep bilaterally over the PFC in children with ADHD. In stimulation-free epochs during deep sleep there was an increase in slow oscillation power, indicating an enhancement of endogenous oscillatory activity as a result of our intervention. The generation of slow oscillations requires larger cell groups to fire highly synchronized, so that the enhancement is not explicable by undirected spreading current after stimulation cessation. We assume that a synchronizing effect of so-tDCS remained present in the post-stimulation intervals (Marshall et al., 2006).

On a behavioral level, behavioral inhibition performance, which is typically impaired in ADHD, was improved. Intrinsic alertness was not influenced by our intervention. We found an overnight speed increase in a motor memory task irrespective of stimulation.

Our findings indicate that slow oscillatory activity during sleep is relevant for sleep-dependent restorative processes with respect to behavioral inhibition or executive functions. Investigations of the role of sleep in attentional functions in ADHD and healthy subjects have been based on descriptive studies (Gruber and Sadeh, 2004) or non-specific restriction protocols (Sadeh et al., 2003; Gruber et al., 2011) so far. Our intervention approach resulting in improved inhibition control through enhancement of slow oscillation power suggests a direct role of slow oscillatory activity during sleep in daytime functioning. Moreover, our results propose SO as a promising somatic marker in the pathophysiology of ADHD (Prehn-Kristensen et al., 2013, 2014; Ringli et al., 2013b). Topographic changes of SWA and functional changes of SO with respect to memory processes have been described earlier in ADHD. Our results demonstrate the significance of slow oscillatory activity for behavioral inhibition processes which are closely intertwined with inattention, impulsivity, and hyperactivity, all cardinal symptoms of ADHD (Barkley, 1997) which limit academic and social outcomes in patients diagnosed with this disorder (Arnsten and Pliszka, 2011). so-tDCS did not influence the (intrinsic) alerting network which is functionally and anatomically disparate from the EF network (Raz, 2004; Raz and Buhle, 2006). Since SO are assumed to originate from the PFC (Massimini et al., 2004; Murphy et al., 2009), our intervention might have stimulated or enhanced processes in these prefrontal cortical regions that are usually altered or defective in ADHD (Arnsten, 2009).

Regarding the motor memory task we found an improvement in reaction times in both the stimulation and the sham condition to a comparable extent. These data suggest a sleep-dependent gain in motor memory, independent of stimulation. However, and in contrast to the behavioral inhibition task, we observed signs of a decrease in motor memory accuracy after the stimulation night. Since this observation is based on the decomposition of a non-significant interaction between session and condition by exploratory *t*-tests, it should be interpreted with caution. In previous studies, healthy children did not benefit from sleep with respect to motor memory, as is observed in healthy adults or children with ADHD (Wilhelm et al., 2008; Prehn-Kristensen et al., 2011b). Since no healthy children were included in this study, it remains speculative whether so-tDCS normalized sleep-dependent motor memory performance in ADHD. However, motor memory data together with the results of the intrinsic alertness task point toward a specific benefit of so-tDCS on inhibition control as a PFC-related function.

so-tDCS in ADHD lead to an improvement of behavioral inhibition and to a possible impairment of procedural memory in our study while declarative memory was enhanced by so-tDCS previously (Prehn-Kristensen et al., 2014). These altered cognitive parameters were influenced toward “healthy” behavior as a result of stimulation. Thus, SO can be considered an important parameter in ADHD, and we suggest their manipulation as a potential therapeutic target.

Several ways of enhancing SWA and SO have been reported. As has been demonstrated for motor areas, slow wave power is dependent on daytime use of the underlying networks (Huber et al., 2006, 2007). Cognitive learning processes increase net synaptic strength, increasing the need for sleep and initial slow wave power (Tononi and Cirelli, 2003, 2006; Huber et al., 2004; Landsness et al., 2009). Thus, one might speculate that cognitive training and improvement of ADHD symptomatology in the daytime would increase the use of executive networks in the PFC. This, in turn, would both lead to increased or normalized slow wave power over prefrontal regions which are usually hypoactive (Ringli et al., 2013a), and a bidirectional effect might emerge. One consequence of improved behavioral inhibition is improved self-regulation with easier initiation of sleep, leading again to better daytime self-regulation repeatedly during development, and might in the long run support PFC maturation (Turnbull et al., 2013). Since (so-)tDCS is currently impractical in a clinical context, and side effects of long-term application are not yet well-investigated, recently developed sensory methods to enhance slow waves seem to be more suitable (Ngo et al., 2013a,b; Bellesi et al., 2014). There is also evidence that sufficiently intense physical activity not only has general health effects but also increases slow-wave sleep in the short-term (Dworak et al., 2008). While methylphenidate is effective during the daytime, the second-line medication atomoxetine increases noradrenergic function independently of the time of day (Sangal et al., 2006; Gamo et al., 2010), a fact which makes this substance interesting for the investigation of sleep in ADHD. SO originate from the PFC (Massimini et al., 2004; Murphy et al., 2009), and the functional effects of pharmacologic manipulation during sleep have not been studied so far.

The interventional design of our study suggests a direct role of SO for behavioral inhibition. However, despite the increase in SO power in stimulation-free intervals, it remains unproven that our specific stimulation mode, sinusoidal SO with a frequency of 0.75 Hz during deep non-REM sleep, was determining our effects. It has been shown in previous work that variation of brain state and stimulation mode leads to dissociable effects (Marshall et al., 2006, 2011). Consequently, extending the design with oscillating and non-oscillating shapes and different intensities of DCS both during wake and sleep to control for general, non-specific stimulation effects, would perhaps have provided more conclusive proof of the specificity of our intervention. Moreover, including healthy control subjects would have been essential for the question of whether so-DCS improves behavioral inhibition in general or specifically in ADHD where it is impaired. In addition, investigating motor memory in healthy children with so-tDCS is necessary to better understand our findings in ADHD. Since reaction times and variability of reaction times in our go/no-go task were influenced by our intervention, but the number of correct responses, commission errors, and omissions were not, although our effects are statistically significant, they are not very comprehensive. Furthermore, not only neurocognitive but also clinical scores would be valuable parameters with respect to the significance of slow oscillations in ADHD, and might be considered in subsequent studies. As a further limitation, correlational analysis of slow oscillation increases, and cognitive performance did not reveal significant effects, probably due to small variance in performance, so that our results should be interpreted cautiously.

In conclusion, we found that slow oscillating transcranial direct current stimulation during early non-REM sleep benefits behavioral inhibition the next morning in children with ADHD. We propose the enhancement of physiological restorative processes during deep sleep, expressed by slow waves (Tononi and Cirelli, 2006) in prefrontal regions as a candidate mechanism. Our results provide further evidence for the significance of sleep for ADHD and daytime cognitive functioning.

## Author contributions

Study conception and design: MTM, APK, MM, RG, LB. Acquisition of data: MTM, FT. Analysis and interpretation of data: MTM, APK, FT, LB. Writing manuscript: MTM, APK, RG, LB.

## Funding

This study was supported by a grant of the German research foundation (SFB 654, Plasticity and Sleep). The funders had no role in study design, data collection, analysis, preparation of the manuscript, or the decision to publish it.

### Conflict of interest statement

The authors declare that the research was conducted in the absence of any commercial or financial relationships that could be construed as a potential conflict of interest.
